# Design and validity of a questionnaire to assess sexuality in pregnant women

**DOI:** 10.1186/1742-4755-6-12

**Published:** 2009-07-29

**Authors:** Cibele VC Rudge, Iracema MP Calderon, Adriano Dias, Gerson P Lopes, Angélica P Barbosa, Izildinha Maestá, Jon Øyvind Odland, Marilza VC Rudge

**Affiliations:** 1 Institute of Community Medicine, Tromso, Norway; 2 Department of Obstetrics and Gynecology, Botucatu Medical School, São Paulo State University (UNESP), Brazil; 3 Department of Obstetrics and Gynecology of Botucatu Medical School, São Paulo State University (UNESP), Brazil; 4 Research Group Council – Botucatu Medical School (UNESP), Brazil; 5 Department of Sexology, Mater Dei Hospital, Belo Horizonte, MG, Brazil; 6 Institute of Community Medicine, University of Tromsø, Norway

## Abstract

**Background:**

A review of validated methods for assessing female sexual dysfunction and a review of male and female sexual dysfunction did not refer to any specific questionnaire for evaluating sexuality during pregnancy. A study was performed at the Obstetrics and Gynecology Department of Botucatu Medical School, São Paulo State University, Brazil to design and validate a pregnancy sexuality questionnaire, the Pregnancy Sexual Response Inventory (PSRI).

**Methods:**

Women with a singleton pregnancy between 10 and 35 weeks of gestation were randomly recruited. There were five phases in the development of the PSRI: (1) item selection; (2) item development; (3) determination of internal consistency, reliability and convergence; (4) content validity; and (5) determination of inter-interviewer reliability. Internal consistency and reliability were evaluated using Cronbach's alpha. Inter-interviewer reliability was assessed by evaluating the responses of 18 academics at various institutions, using Kappa Index and Student t test.

**Results:**

Good internal consistency and reliability were obtained (Cronbach's alpha coefficient = 0.79). Among the 18 academics, 13 totally agreed (K = 1.0), three partially agreed (K = 0.67) and two disagreed (K = 0.33) with the proposed questions. Comparisons of the mean PSRI domain scores made between the primary investigators and the other interviewers showed no significant differences in all domains (p > 0.05).

**Conclusion:**

PSRI is a new validated instrument for evaluating sexuality and sexual activity and related health concerns during pregnancy.

## Background

Pregnancy and birth mark a very special period in a woman's life. It is a time of physical and psychological changes that commonly impact women's physical well-being, mood, relationships and sexuality [1]. Many studies exploring sexual activity and correlated factors during pregnancy were performed more than two decades ago, and they focused primarily on genital response. In addition, they were published before the most important concepts of sexual satisfaction and distress began to be considered by researchers in this field.

A variety of brief self-report measures have been developed for assessing female and male function across a variety of sexual domains (e.g., sexual desire, arousal, orgasm and satisfaction). These brief self-report measures have been shown to have high reliability and validity and to be sensitive to treatment interventions. A review of validated methods for assessing female sexual dysfunction [2] and a review of male and female sexual dysfunction [3] did not refer to any specific questionnaire for evaluating sexuality during pregnancy.

Changes in attitudes towards sexuality during pregnancy, the different sexual responses proposed by Basson [4], the limitations of methodological flaws (i.e., small sample sizes, unrepresentative samples, and retrospective data), and inconsistencies in the results from published studies may limit the relevance of many studies [5].

Health care professionals report a number of barriers when inquiring about their patients' sexual functioning, including concerns about embarrassing their patients, perceptions that patients lack confidence in their professional knowledge of sexuality, time constraints, and personal discomfort in talking about sex [6,7].

Over the past few years, there has been growing interest among healthcare providers in quantifying female sexual dysfunction during pregnancy. Obstetricians have become interested not only in the direct effects of pregnancy but also in the impact of pregnancy or the adaptation to pregnancy has on the woman's overall well-being.

An extensive literature search on the prevalence and predictors of female sexual dysfunction reported no valid assessment for pregnancy and the postpartum period [8]. Barclay [9] developed the Pregnancy and Sexuality Questionnaire (PSQ), a validated instrument for studying sexual relations between partners during pregnancy, although they did not list the specific items included in their questionnaire within their article. Due to the lack of access to the only validated instrument, we created a new instrument to assess the impact of pregnancy on sexuality and sexual activity that was based on the PSQ. An additional goal was to further the state of the evidence through expanding the assessment to include questions about sexual satisfaction and distress, as proposed by the Second International Consultation on Sexual Dysfunctions [10].

The present investigation was undertaken to design and validate a Pregnancy Sexual Response Inventory (PSRI) to evaluate changes in sexuality during pregnancy, that was brief, broad in scope, useful for both low and high-risk pregnancies, validated, and available in full text.

## Methods

A cross-sectional study was conducted at the Obstetrics and Gynecology Department of Botucatu Medical School, São Paulo State University (UNESP), Brazil to design and validate a questionnaire for assessing women's sexual attitudes and practices during pregnancy. It utilized a public hospital clinic for patients from diverse socioeconomic backgrounds, including women both with and without private health insurance. Approval for the study was given by the local Institutional Research Bureau (IRB), and a written statement of informed consent was obtained from all participants prior to interview.

All pregnant women attending prenatal consultations at Botucatu Medical School between July 2004 and December 2005 were eligible. Women with a singleton pregnancy between 10–35 weeks of gestation, approximately equally distributed across the three trimester of pregnancy, were approached to participate in this study. Women were not eligible to participate in the study if they had been diagnosed with medical or obstetric conditions that made sexual intercourse inadvisable (e.g., placenta previa, antepartum hemorrhage or threatened preterm labor). No sample-size calculations were specifically considered for the assessment of sexual behavior and activity during pregnancy, because this was not a hypothesis-driven study but was mainly descriptive in nature. The participants were assured that the survey was anonymous and that their responses would be kept strictly confidential. Participants were also informed that they would be helping to improve knowledge of sexuality during pregnancy among Brazilian women. The semi-structured interviews were all performed by the same female researcher (CVCR) and took place in an undisturbed room at the prenatal care clinic. Each participant was interviewed individually immediately after recruitment.

There were five phases in development of the PSRI: (1) item selection; (2) item development; (3) determination of internal consistency, reliability and convergence; (4) content validity; and (5) determination of inter-interviewer reliability. For item selection an α-questionnaire given to 42 subjects, to understand how Brazilian women perceive their sexuality, consisted of 45 items that were developed using as references the Brazilian Sexual Life Study[11], the Female Sexual Function Index (FSFI)[12], and the Pregnancy and Sexuality Questionnaire (PSQ) [9]. Both qualitative and quantitative items were included. The aim of this initial phase was to meet basic established criteria, i.e., to be clear and understandable, to provide comprehensive response choices, and to be relatively simple to administer. The domains assessed comprised socio-demographic status, perceptions of sexuality, and sexual behavior during pregnancy. The socio-demographic details gathered were: age, marital status, education level, occupation, obstetric history, religious affiliation, drug and alcohol abuse, and smoking. The domain of perceptions of sexuality covered typical sexual activities: tenderness, condom use during pregnancy, and importance of sex life. The domain of sexual behavior during pregnancy were items including frequency of sexual intercourse, sexual satisfaction, arousal, sexual difficulties and dysfunction, sexual desire, orgasm, dyspareunia, beginning of sexual intercourse and the pregnant woman's opinion about her partner's sexual response, sexual satisfaction and difficulties during pregnancy.

For item development (phase 2), a β-questionnaire was formulated. This utilized a 38-item interview based on the results of phase 1. Unlike the α-questionnaire, only one response was accepted for each question, and responses were limited to three possible answers. It was designed to elicit information about demographic characteristics and sexuality before and during pregnancy. Demographic characteristics assessed were: maternal age, gestational age, partnership status, socioeconomic status (i.e., education level and occupation), alcohol use, drug abuse, smoking during pregnancy, pregnancy planning and condom use. The questions relating to sexuality and sexual activity that were included as items in the three initial PSRI domains were categorized into nine domains: frequency of sexual activity; sexual satisfaction; arousal; sexual difficulties and dysfunction; sexual desire; orgasm; dyspareunia; beginning of sexual intercourse; pregnant woman's opinion about partner's sexual response, including the man's satisfaction; and difficulties during pregnancy.

Sixty-three pregnant women were recruited to participate in the evaluation of internal consistency (phase 3). All responded to the β-questionnaire.

In the fourth phase, content validity, the 38-item questionnaire resulting from the phase 3 testing was sent by mail to an independent expert panel of 25 PhD level academic experts in pregnancy and sexuality. Each candidate item was assessed for clinical relevance, breadth of scope, ease of understanding, language level, brevity for use in a busy prenatal care clinic, and adequacy as an instrument for evaluating the influence of pregnancy on the female sexual response.

Based on the results of this process, 26 candidate items were selected for the reliability evaluation (phase 5). Ten doctoral students in four different cities without any previous training each applied the PSRI to 10 subjects. Their results were compared to those of the Botucatu interviewees. Internal consistency, which is a correlational determination of the goodness of fit of the items within a domain, was measured on a scale of 0–1. Reliability is a measure of the relatedness of items within each factor.

The final PSRI was divided into 10 domains, eight of them relating to the woman's feelings and two to her perceptions of her partner. All domains included items on possible distress, as this concept is necessary to investigate sexual dysfunction. The eight domains of female feelings included: (a) Frequency, a three-item scale that assessed frequency of sexual intercourse relating to pregnancy; (b) Desire: a three-item scale that assessed the frequency of desire before and during pregnancy and the frequency of participation in sexual activity; (c) Arousal: a three-item scale that assessed the quality of sexual activity before and during pregnancy; (d) Orgasm: a three-item scale that assessed the frequency of orgasm before and during pregnancy; (e) Pleasure: a three-item scale that assessed the enjoyment of sex life before and during pregnancy; (f) Dyspareunia: a two-item scale that assessed pain during sexual intercourse before and during pregnancy; (g) Intercourse initiation: a three-item scale that assessed the start of participation in sexual activity before and during pregnancy; and (h) Female difficulties: a two-item scale that assessed any female sexual difficulties before and during pregnancy. The woman's perception of her partner's sexuality included: (i) Male sexual pleasure: a three-item scale that assessed the female view of male pleasure before and during pregnancy; and (j) Male sexual difficulties: a two-item scale that assessed the female view of male sexual difficulties before and during pregnancy.

### Statistical Analyses

The first series of evaluations was performed on an item-by-item basis in order to obtain items with adequate properties and clinical relevance for the final inventory. The internal consistency and reliability of the PSRI items within each factor were evaluated using Cronbach's alpha coefficient [13,14]. The inter-interviewer reliability was assessed by means of the Kappa Index[15]. Reproducibility was evaluated using the Student t-test [16]. All data were analyzed using SAS statistical software version 8.2 [17].

## Results

Forty-two pregnant women participated in phase 1. This α questionnaire accepted more than one answer for each question and was not drawn up in sequential order. We reviewed the univariate frequencies and calculated Cronbach's alpha coefficient to assess internal consistency and reliability [13,14]. The alpha coefficient was -3.2, which indicated low reliability.

We then developed a β-version of the questionnaire (phase 2) building on the information acquired from this first effort. Responses of 63 women were analyzed. We evaluated (stage 3) the univariate frequencies of responses and calculated Cronbach's alpha to establish the internal consistency reliability. Demographic characteristics were excluded from the Cronbach's alpha coefficient calculation. Cronbach's alpha coefficient for the β-version of the PSRI was 0.79.

Content validity is an essential methodological consideration in developing a questionnaire. It is concerned with the adequacy with which questions reflect the domains that are being measured. As the PSRI is a new questionnaire, it was important that its credibility be established. Of the 25 experts whose evaluation was requested with regard to its validity and reliability 18 (72%) responded. The specific questions asked and the responses are shown in Table 1. Among the 18 academics who returned the survey, 13 totally agreed (K = 1.0), three partially agreed (K = 0.67) and two disagreed (K = 0.33). K indices of more than 0.5 were considered to be good correlations. We used the comments and critical reviews to revise the PSRI with regard to identify partner characteristics (partner age, partner schooling level, stability of partner relationship and occupation) and question order. We did not implement certain suggestions, including the recommendations that open-ended questions be used (i.e., where, why, how) and that more than one answer be allowed for each question because we had already eliminated these possibilities when moving from the alpha to the beta version of the questionnaire.

**Table 1:** Content validity of PSRI

**Items**	**Yes (%)**	**No (%)**	**Some (%)**
1 – Are the questions in the PSRI broad in scope?	72.2	16.6	11.1
2 – In your opinion, are more questions needed in order to cover all the domains?	27.7	72.2	--
3 – Will the questions be easy for OG or the health team to understand?	83.4	16.6	0
4 – Are the words clear?	88.9	11.1	0
5 – Are there enough questions in the PSRI regarding screening for female sexual dysfunction at prenatal consultations?	88.9	11.1	--
6 – Are there enough questions in the PSRI for evaluating the influence of pregnancy on female sexual response?	88.9	11.1	--

Kappa index: 0.33 to 1.00.

Reliability (phase 5) refers to the stability of measurement exhibited when a questionnaire is administered at different times (test-retest reliability) or by different people (inter-interviewer reliability). Because the PSRI is a semi-structured interview, inter-interviewer reliability is relevant to its development. The test-retest procedure is difficult to assess in relation to pregnancy because our prenatal care routine establishes four-week intervals between visits, and this interval is large enough for the subject's behaviour to change naturally as the pregnancy progresses. A Cronbach's alpha coefficient of 0.73 was obtained for the 38-item PSRI, indicating good internal consistency and reliability. A subsequent calculation excluding demographic items 1–12 was conducted; we found a Cronbach's alpha coefficient of 0.79 for the remaining 26 items.

Comparisons of the mean PSRI domain scores made between the main researcher and other interviewers showed no significant differences in all domains (p > 0.05) (Table 2).

**Table 2:** Comparisons* of mean PSRI domain scores between the main researcher and other interviewers

**Domains**	Rudge, CVC	***Other interviewers***	p-value*
Frequency	1.8848 (± 0.6946)	1.9497 (± 0.7677)	0.617**
Desire	1.9787 (± 0.9189)	1.9667 (± 0.9153)	0.760**
Arousal	2.3252 (± 0.6465)	2.3700 (± 0.7199)	0.908**
Orgasm	2.1920 (± 0.8102)	2.3800 (± 0.7886)	0.212**
Satisfaction	2.5344 (± 0.6609)	2.2700 (± 0.7939)	0.065**
Dyspareunia	1.6260 (± 0.4858)	1.5556 (± 0.5207)	0.468**
Intercourse start	2.4667 (± 0.7063)	2.3086 (± 1.1070)	0.379**
Female difficulties	1.5556 (± 0.6146)	1.6081 (± 0.6674)	0.683**
About partners			
Male sexual satisfaction	2.4538 (± 0.6733)	2.2604 (± 0.8996)	0.217**
Male sexual difficulties	1.7480 (± 0.4360)	1.8500 (± 0.3589)	0.187**

*T-test; **: non significant.

The final PSRI model is shown in the Additional file 1 and consists of a pool of 38 items that address aspects of female sexual function during pregnancy.

## Discussion

This study documents the structure, internal consistency, reliability, content validity and inter-interviewer reliability of the Pregnancy Sexual Response Inventory (PSRI). This is a new questionnaire for evaluating sexuality and sexual activity during pregnancy. It was designed to be a clinical assessment instrument for addressing health concerns regarding to sexuality during pregnancy. Furthermore, this instrument is intended for use as a screening tool for sexual disorders during pregnancy. It is designed to be used by health care providers to assess the quality of pregnant women's sexual lives and to determine if referral to a sexologist is necessary. The PSRI was developed over a series of stages, including preselection of the initial items, pretesting on volunteer pregnant women and then validation of the internal consistency reliability and content validity by a panel of expert consultants. The results from the present investigation demonstrated that the PSRI has good internal consistency and reliability over its entire scale (Cronbach's α = 0.79) and is suitable for use in assessing sexual function during pregnancy in obstetric clinical samples.

The test-retest reliability was difficult to assess during pregnancy because of the typical interval between prenatal visits. A two-week period would have been ideal: short enough for the woman's behavior not to undergo the natural changes over the course of the pregnancy, but long enough for them not to remember the responses that they had given on the first occasion [9]. Inter-interviewer reliability was examined, and the results confirmed that the instrument was reliable. In all domains, the results showed no significant differences between the main researcher and other interviewers. To minimize bias, other interviewers included both male and female researchers in the last phase, all doctoral students, but not all physicians (e.g., physiotherapists, nurses, and psychologists). These results are relevant as clinically validated questionnaires to be used in Brazil and other Portuguese-speaking areas after review by specialized teams in cultural translation and adaptation. We also developed a prototype English version, but, due to cultural differences, further modifications specific to each country may be necessary. If a translation is created and back translated, a new validation study should be done [18].

One limitation to this tool is the incorporation of questions around pre-pregnancy sexual function. Although these items are essential for comparison, recall bias may challenge the validity of these responses. Therefore, we suggest that this questionnaire can optimally be applied before and during pregnancy to limit recall bias. Further studies are necessary to determine the extent to which recall bias may limit results validity.

It is important that healthcare providers in the field of obstetrics are able to counsel their patients on the emotional aspects related to sexual alterations during pregnancy and sexual aspects during pregnancy. [19]. Although women feel that attention should be given to sexuality and sexual activity during pregnancy and wish to receive more information around these issues, this is rarely discussed between pregnant women and their physicians [5,19]. The availability of a brief, valid, reliable and gender-specific self-reported questionnaire for monitoring sexual functioning may diminish these concerns [7].

As a next step, we plan to develop validated thresholds that should be used in prenatal care to indicate referral to a sexologist.

## Conclusion

In summary, the PSRI is a 38-item (12 demographic characteristics and 26 sexual behavior/activity) clinical instrument providing a brief, semi-structured interview for assessing the impact of pregnancy on sexuality. Internal consistency and content validity testing of questionnaire items by an expert panel was followed by an assessment of inter-interviewer reliability. Since the PSRI is a clinically validated questionnaire that is easy to administer, its value as a research instrument should be investigated.

## Competing interests

The first author is recipient of a doctoral PDEE fellowship from the Brazilian Federal Agency for Graduate Studies (CAPES, Ministry of Education). The authors declare they have no competing financial interests.

## Authors' contributions

CR carried out the conception, study design, acquisition of data, and draft the manuscript. IC participated in its design and coordination and helped to draft the manuscript. AD participated in the design of the study and performed the statistical analyses. IM carried out the acquisition of the data and draft the manuscript. AB carried out the acquisition of the data and participated in statistical analyses. GL carried out the literature review and participated in the study design. JO carried out a critically review for important intellectual content. MR: carried out the draft of manuscript and the final approval of the version to be published. All authors read and approved the final manuscript.

## Supplementary Material

Additional file 1 **Items comprising the final version of the PSRI.** Click here for file
