# Supplementary material for: Design and validity of a questionnaire to assess sexuality in pregnant women
Source: Reprod Health. 2009 Jul 29;6:12. doi: 10.1186/1742-4755-6-12 (PMC2724386; doi:10.1186/1742-4755-6-12)
Supplement: Additional file 1 — Items comprising the final version of the PSRI. [file 1742-4755-6-12-S1.doc]

**Additional file 1 -** Items comprising the final version of the PSRI

| **I - Demographic characteristics** | |
| --- | --- |
| 1- Mother’s age:  Partner’s age: | 2- Gestational age: |
| 3- Partnership status?  (1) married/ living together  (2) single  (3) other_______ | 4- Education Level:  (1) basic level  (2) high school  (3) college/university |
| 5- Religion  (1) Catholic  (2) Evangelical  (3) other_________ | 6- Do you work?  (1) no  (2) yes, and I currently have a job  (3) yes, but I am currently unemployed |
| 7- Do you have children?  () no  () just one  () two or more | 8- Do you smoke?  () yes, often or very often  () yes, but just sometimes  () no |
| 9- Do you drink?  () yes, often or very often  () yes, but just sometimes  () no | 10- Do you use illicit drugs?  () yes, often or very often  () yes, but just sometimes  () no |
| 11- Did you plan your pregnancy?  () yes  () no | 12- Do you use condoms?  () no  () yes, but I stopped before I became pregnant  () yes, often or very often |
| **II- Sexual behavior/ activity before and during pregnancy** | |
| 13-In your opinion, did your frequency of sexual activity change after you became pregnant?  (1) yes, it decreased  (2) no, it stayed the same  (3) yes, it increased | 14a- Before your pregnancy, how many times per week did you have sexual intercourse?  (1) none  (2) 1-2 times  (3) 3 or more times |
| 14b- In the first trimester of pregnancy, how many times per week did you have sexual intercourse?  (1) none  (2) 1-2 times  (3) 3 or more times | 14c- At present, how many times per week are you having sexual intercourse?  (1) none  (2) 1-2 times  (3) 3 or more times |
| 15a- How would you rate your sex life before you became pregnant (0 = lowest, 10 = highest)?  (1) 0-3  (2) 4-7  (3) 8-10 | 15b- How would you rate your sex life at present (0 = lowest, 10 = highest)?  (1) 0-3  (2) 4-7  (3) 8-10 |
| 16a- How do you think your partner would rate his sex life before you became pregnant (0 = lowest, 10 = highest)?  (1) 0-3  (2) 4-7  (3) 8-10 | 16b- How do you think your partner would rate his sex life at present (0 = lowest, 10 = highest)?  (1) 0-3  (2) 4-7  (3) 8-10 |
| 17a- Were you satisfied with your sexual life before you became pregnant?  (1) no  (2) somewhat  (3) yes | 17b- Are you satisfied with your sexual life during pregnancy?  (1) no  (2) it was OK I suppose  (3) yes |
| 18a- How would you rate your arousal before your pregnancy?  (1) poor/ very poor  (2) regular  (3) excellent | 18b- How would you rate your arousal during your pregnancy?  (1) poor/ very poor  (2) regular  (3) excellent |
| 19a- Were you having any sexual difficulties before your pregnancy?  (1) yes  (2) no | 19b- Have you been having any sexual difficulties during your pregnancy?  (1) yes  (2) no |
| 20- Do these difficulties distress you?  (1) yes  (2) somewhat  (3) no | 21a- How often did you experience sexual desire before your pregnancy?  (1) a few times a week  (2) once a day  (3) other (depending on the occasion ) |
| 21b- How often have you been experiencing sexual desire during your pregnancy?  (1) a few times a week  (2) once a day  (3) others (depending on the occasion ) | 22- What happened to your sexual desire after you became pregnant?  (1) it decreased  (2) it stayed the same  (3) it increased |
| 23a- How often did you achieve orgasm before your pregnancy?  (1) never/rarely  (2) sometimes  (3) often or very often | 23b- How often have you been achieving orgasm during your pregnancy?  (1) never/rarely  (2) sometimes  (3) often or very often |
| 24a- Did you experience pain during sexual intercourse before you became pregnant?  (1) yes  (2) no | 24b- Have you been experiencing pain during sexual intercourse since you became pregnant?  (1) yes  (2) no |
| 25a-The initiation of intercourse before pregnancy was:  (1) forced, without any desire  (2) partner usually made the first move  (3) spontaneously or spontaneously with stimuli | 25b- The initiation of intercourse during pregnancy is?  (1) forced, without any desire  (2) partner usually makes the first move  (3) spontaneously or spontaneously with stimuli |
| 26a- In your opinion, do you think your partner had any sexual difficulties before your pregnancy?  (1) yes  (2) no | 26b- In your opinion, do you think your partner has been having any sexual difficulty during your pregnancy?  (1) yes  (2) no |
